# The Cyclin B2/CDK1 Complex Conservatively Inhibits Separase Activity in Oocyte Meiosis II

**DOI:** 10.3389/fcell.2021.648053

**Published:** 2021-03-11

**Authors:** Jian Li, Hong-Yong Zhang, Feng Wang, Qing-Yuan Sun, Wei-Ping Qian

**Affiliations:** ^1^Department of Reproductive Medicine, Peking University Shenzhen Hospital, Shenzhen Peking University-The Hong Kong University of Science and Technology Medical Center, Shenzhen, China; ^2^State Key Laboratory of Stem Cell and Reproductive Biology, Institute of Zoology, Chinese Academy of Sciences, Beijing, China; ^3^Fertility Preservation Lab, Reproductive Medicine Center, Guangdong Second Provincial General Hospital, Guangzhou, China; ^4^Guangdong Key Laboratory of Male Reproductive Medicine and Genetics, Institute of Urology, Peking University Shenzhen Hospital, Shenzhen PKU-HKUST Medical Center, Shenzhen, China

**Keywords:** cyclin B2, separase, meiosis II, oocyte, mouse 36

## Abstract

Recently, we have reported that the cyclin B2/CDK1 complex regulates homologous chromosome segregation through inhibiting separase activity in oocyte meiosis I, which further elucidates the compensation of cyclin B2 on cyclin B1’s function in meiosis I. However, whether cyclin B2/CDK1 complex also negatively regulates separase activity during oocyte meiosis II remains unknown. In the present study, we investigated the function of cyclin B2 in meiosis II of oocyte. We found that stable cyclin B2 expression impeded segregation of sister chromatids after oocyte parthenogenetic activation. Consistently, stable cyclin B2 inhibited separase activation, while introduction of non-phosphorylatable separase mutant rescued chromatid separation in the stable cyclin B2-expressed oocytes. Therefore, the cyclin B2/CDK1 complex conservatively regulates separase activity *via* inhibitory phosphorylation of separase in both meiosis I and meiosis II of mouse oocyte.

## Introduction

Mammalian oocyte meiosis consists two continuous cell divisions, concomitantly, the chromosomes undergo two rounds of consecutive segregations, with segregation of homologous chromosomes in the first meiotic division and segregation of sister chromatids in the second meiotic division (Petronczki et al., 2003). To accomplish this orderly and accurate transmission of the duplicated genome into haploid gametes, the homologous chromosomes synapsed and paired during prophase stage to facilitate subsequent chiasmata formation by the action of cohesin, a ring-like four-subunit complex (Gruber et al., 2003; Nasmyth and Haering, 2005). Cohesin ties the sister chromatids together by its localizations at centromeres and chromosome arms, and the removal of arm cohesin allows the resolution of chiasmata and segregation of homologs in meiosis, while the removal of centromeric cohesin allows the segregation of sister chromatids in meiosis II (Terret and Jallepalli, 2006). In mouse oocyte, the cohesin loss is attributed to separase-dependent cleavage of REC8 subunit, which also requires the phosphorylation of REC8 (Katis et al., 2010; Chambon et al., 2013). To achieve the segregation of homologs only in meiosis I, the centromeric cohesin is under protection in meiosis I by Shougoshin2-associated PP2A recruitment mechanism (Kitajima et al., 2006; Riedel et al., 2006).

Previously, separase activity was known to be mutually inhibited by binding to securin and cyclin B1/CDK1 complex (Zou et al., 1999; Lee and Orr-Weaver, 2001; Stemmann et al., 2001; Petronczki et al., 2003; Holland and Taylor, 2006). In a recent study, we demonstrated that the cyclin B2-associated CDK1 activity was involved in the separase regulation in oocyte meiosis I, and expression of non-degradable cyclin B2 completely prevented the segregation of homologs in the cyclin B1-null oocytes, arresting the oocytes at metaphase I with the normal anaphase-promoting complex or cyclosome (APC/C)-dependent degradation of securin (Li et al., 2019). Cyclin B1 and cyclin B2 are two B-type cyclins, both can bind to CDK1 to activate maturation-promoting factor (MPF), which is required for oocyte meiotic resumption. Deletions of cyclin B1 and cyclin B2 together in mouse oocytes results in failure of meiotic resumption. Interestingly, cyclin B2 is important for meiotic resumption and it can compensate for cyclin B1 during oocyte meiosis I (Gui and Homer, 2013; Li et al., 2018; Daldello et al., 2019), even though cyclin B1 caught the attention of researchers in the past many years. Indeed, cyclin B1 is indispensable for meiosis II entry because the cyclin B1-deleted oocytes entered interphase-like stage after extrusion of the first polar body; moreover, expression of exogenous cyclin B2 rescued this phenotype to allow the meiosis II entry (Li et al., 2018). We now wonder the function of cyclin B2 on chromatid separation in oocyte meiosis II. In metaphase II (MII) eggs, expression of either non-degradable securin, or non-degradable cyclin B1 blocked the sister chromatid segregation (Madgwick et al., 2004), reflecting the roles of securin and cyclin B1 on separase regulation in meiosis II. However, whether cyclin B2/CDK1 complex has the ability to inhibit separase activity during meiosis II remains unknown.

Here, we investigated the function of cyclin B2 in sister chromatid segregation. We found that stable cyclin B2 expression induced the failure of oocyte activation, characterized by the absence of pronucleus formation. We further showed that sister chromatids did not separate in the cyclin B2-expressed oocytes due to the lack of separase activity, while CDK1-resistant phosphorylation site mutant separase (PM-separase) introduction rescued the segregation of sister chromatids. Thus, we propose that the cyclin B2/CDK1 complex conservatively inhibits separase activity in oocyte meiosis II.

## Results

### Stable Cyclin B2 Expression Prevents Pronucleus Formation After Parthenogenetic Activation in Mouse Oocytes

**FIGURE 1:**
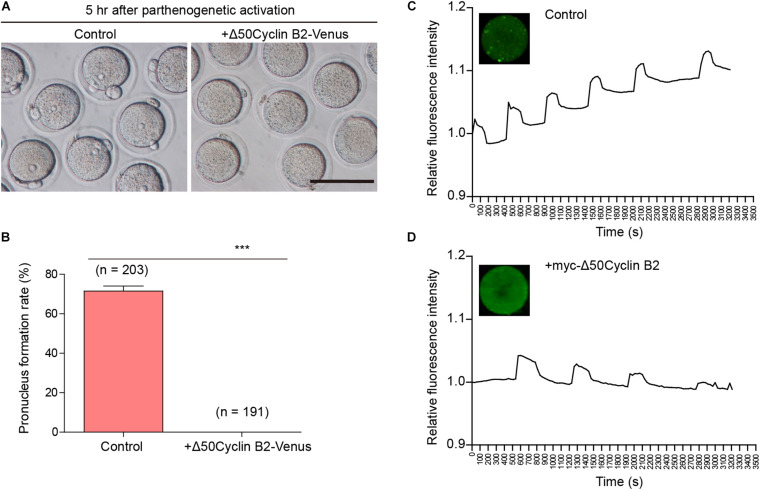
Stable cyclin B2 expression led to the failure of parthenogenetic activation in mouse oocytes. **(A)** Observation of pronucleus formation in the control and Δ50cyclin B2-Venus-expressed MII oocytes after PA. The pronucleus formation was observed 5 h after PA. Scale bar represents 100 μm. **(B)** Rates of pronucleus formation in the control and Δ50cyclin B2-Venus-expressed MII oocytes. The concentration of Δ*50cyclin B2-Venus* mRNA used for injection was 500 ng/μl. Data are presented as mean + SEM. ***P < 0.0001 by Student’s *t*-test. The numbers of oocytes used (*n*) are shown. **(C)** The intracellular Ca^2+^ oscillations in the control MII oocytes during PA. **(D)** The intracellular Ca^2+^ oscillations in the stable cyclin B2-expressed MII oocytes during PA.

We recently described that the cyclin B2/CDK1 complex was involved in the regulation of homologous chromosome separation by inhibiting separase activity during mouse oocyte meiosis I, and that expression of non-degradable cyclin B2 arrested the oocytes at metaphase I (Li et al., 2019). We wonder whether cyclin B2 regulates the separation of sister chromatids in meiosis II. To address this point, the Δ*50cyclin B2-Venus* mRNA was introduced into the MII oocytes collected from the oviducts 14 h after HCG injection, then the oocytes were transferred into Ca^2+^-free SrCl_2_-CZB medium for parthenogenetic activation (PA) after 2 h incubation in KSOM medium to examine whether the oocytes could be activated as the control oocytes. We found that no pronucleus formed in the stable cyclin B2-expressed oocytes 5 h after PA (Figures 1A,B), while most of the control oocytes had formed pronuclei with the second polar body extruded (Figures 1A,B). We checked the intracellular Ca^2+^ oscillations, which is required for oocyte activation, in the control and stable cyclin B2-expressed MII oocytes during PA, we found that the Ca^2+^ oscillations was initiated normally, but showed longer cycle and decreased intensity in the stable cyclin B2-expressed oocytes (Figures 1C,D and Supplementary Movies 1, 2). These results indicated that the stable cyclin B2 prevented MII oocyte activation.

### Stable Cyclin B2 Expression Prevents Separation of Sister Chromatids in Meiosis II

**FIGURE 2:**
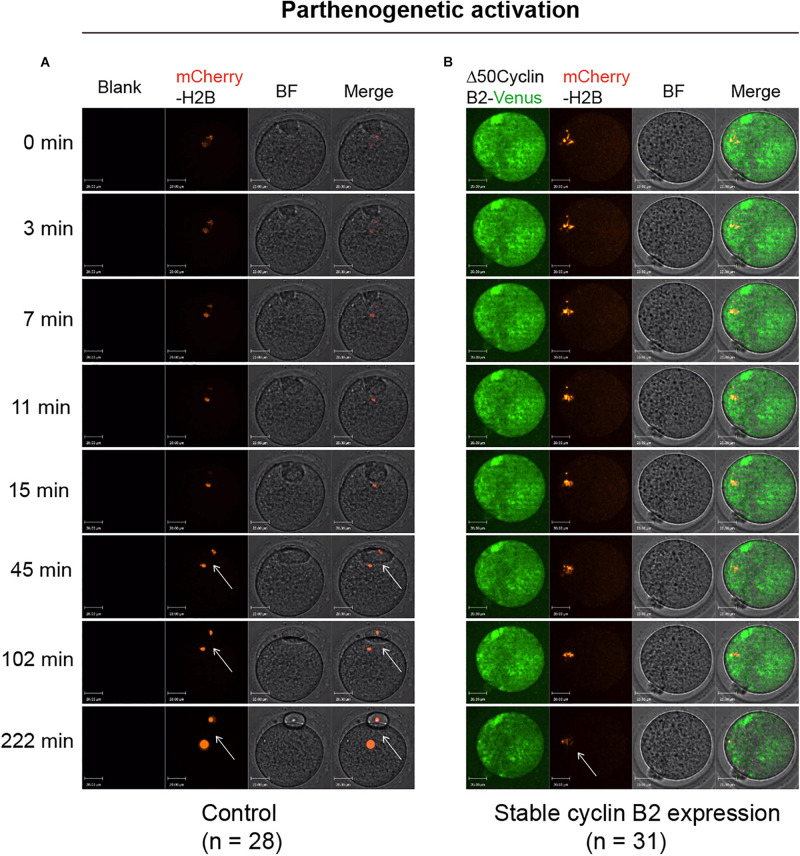
Representative time-lapse confocal images for mCherry-H2B in the control and Δ50cyclin B2-Venus-expressed oocytes. In the control group, the MII oocytes collected from oviducts were injected with *mCherry-H2B* mRNA **(A)**. In the experimental group, the Δ*50cyclin B2-Venus* and *mCherry-H2B* mRNAs were co-injected into the MII oocytes **(B)**. After 2 h incubation in KSOM medium, the oocytes were transferred into Ca^2+^-free CZB medium for parthenogenetic activation under live cell imaging system. The arrows pointed out the separated sister chromatids in **(A)** and unseparated sisters in **(B)**. The concentrations of *mCherry-H2B* and Δ*50cyclin B2-Venus* mRNAs used for injection were 200 and 500 ng/μl, respectively. Scale bars represent 20 μm in **(A,B)**.

To identify the effect of stable cyclin B2 expression on the separation of sister chromatids, we used a mCherry-H2B probe to label chromosomes to observe the chromosome separation after PA in live cells. The Δ*50cyclin B2-Venus* and *mCherry-H2B* mRNAs were co-injected into the MII oocytes 2 h before PA. In the control oocytes, the sister chromatids separated soon after PA (Figure 2A and Supplementary Movie 3), on the contrary, the sisters did not separate in the stable cyclin B2-expressed MII oocytes even with the extended observation (Figure 2B and Supplementary Movie 4). Therefore, we concluded that the presence of non-degradable cyclin B2 prevented the disjunction of sister chromatids in the second meiotic division in oocytes, in other words, the degradation of cyclin B2 was required for sister chromatid separation in oocyte meiosis II.

### Stable Cyclin B2 Expression Inhibits Separase Activity in Meiosis II

**FIGURE 3:**
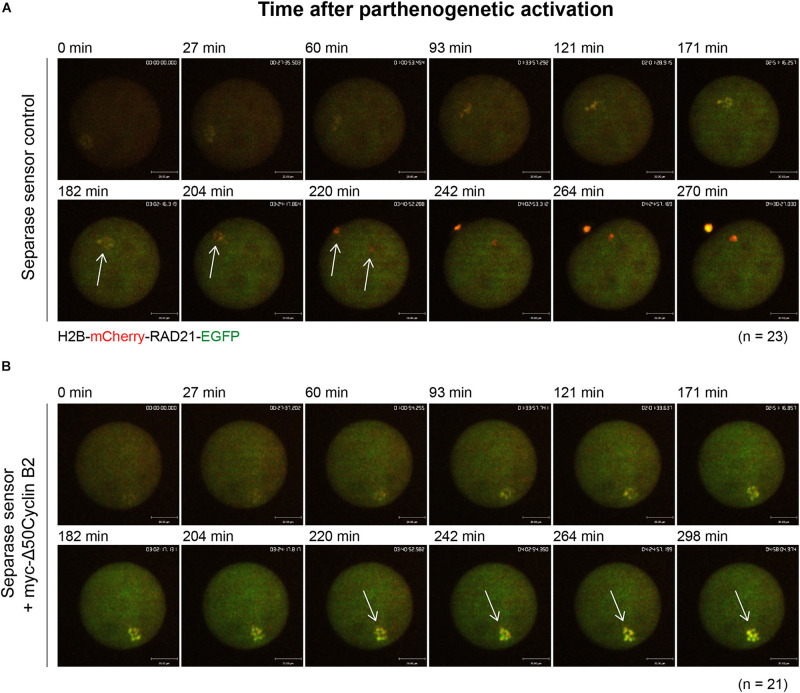
Stable cyclin B2 expression inhibited separase activation in meiosis II. **(A)** Representative time-lapse confocal images for separase sensor in the control oocytes after PA. The concentration of separase sensor mRNA used was 200 ng/μl. The arrows indicated the changes of separase sensor during disjunction of sister chromatids. **(B)** Representative time-lapse confocal images for separase sensor in the myc-Δ50cyclin B2-expressed oocytes after PA. The arrows indicated the unchanged signal of separase sensor with non-disjunction of sisters. The concentrations of separase sensor and *myc-Δ50cyclin B2* mRNAs used for injection were 200 and 500 ng/μl, respectively. The mRNAs were injected into MII oocytes 2 h before PA. The numbers of oocytes used (*n*) are shown. Scale bars represent 20 μm in **(A,B)**.

Taken the fact that separase activity is responsible for chromosome separation, and cyclin B2/CDK1 complex inhibits separase activity in meiosis I, we wonder if it also acts in the same way in meiosis II. To test this hypothesis, we applied a separase sensor worked well before (Li et al., 2019, 2020) to detect the separase activity directly in oocyte meiosis II after PA. In the control oocytes, we observed a distinct fluorescent change of separase sensor, which turned into mCherry signal only after chromatid separation during PA as expected (Figure 3A and Supplementary Movie 5). In contrast, the separase sensor remained with no significant change from the beginning to the end in the stable cyclin B2-expressed oocytes (Figure 3B and Supplementary Movie 6). This result suggested that the cyclin B2/CDK1 complex conservatively inhibited separase activity in oocyte meiosis II.

### Non-phosphorylatable Separase Induces Sister Chromatid Separation in the Stable Cyclin B2-Arrested MII Oocytes After PA

**FIGURE 4:**
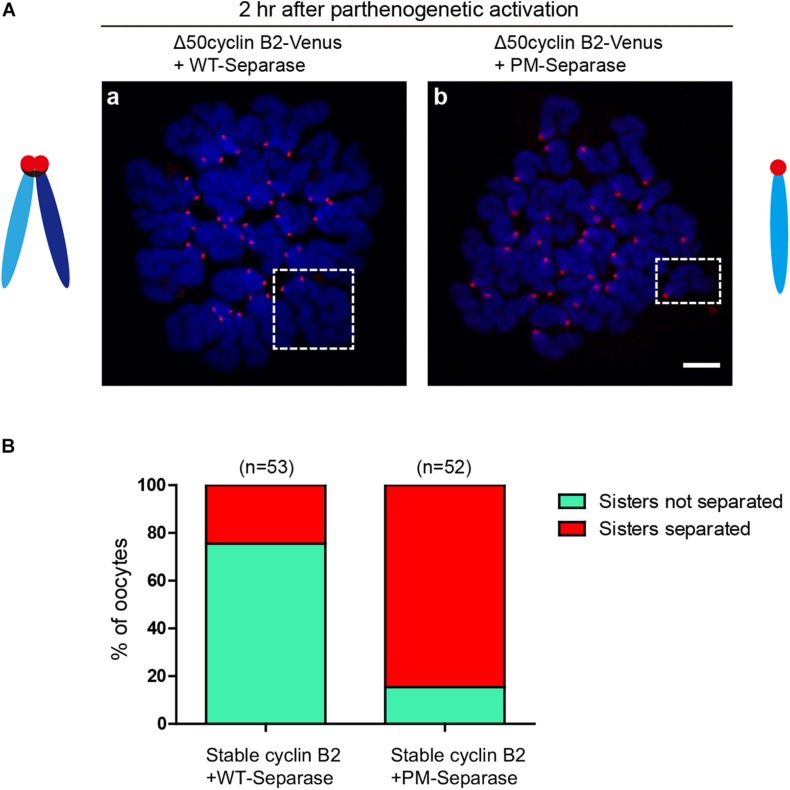
Non-phosphorylatable separase rescued the chromosome non-disjunction in the stable cyclin B2-expressed MII oocytes. **(A)** Chromosome spreads for the oocytes co-expressing Δ50cyclin B2-Venus and WT-separase (a), and co-expressing Δ50cyclin B2-Venus and PM-separase (b). The selected areas in (a,b) are the representative dyad (sisters not separated) and univalent (sisters separated), respectively. Centromere and DNA were stained with ACA antibody and DAPI, respectively. ACA, anti-centromeric antibodies. In the diagrams, light blue and dark blue represent sister chromatids, black represents cohesin, and red represents the centromere, respectively. **(B)** Statistical percentage histogram for oocytes with sisters not separated or separated in the two groups in **(A)**. Green represents the proportion of sisters not separated, and red represents the proportion of sisters separated. The numbers of oocytes used (*n*) are shown.

To confirm the inhibitory role of cyclin B2/CDK1 complex in separase activity by phosphorylation in meiosis II, the phosphorylation site (S1121)-mutated mouse separase (PM-separase) was introduced to rescue the separation of chromatids in the stable cyclin B2-arrested MII oocytes after PA; as the control, the wildtype mouse separase (WT-separase) was introduced for co-expression. In most of the stable cyclin B2 and WT-separase co-expressed oocytes (75.5%), the sister chromatids were still maintained in pairs (Figures 4Aa,B); whereas in the majority of oocytes (84.6%) co-expressing stable cyclin B2 and PM-separase, the sister chromatids had separated clearly 2 h after PA (Figures 4Ab,B). This result confirmed that cyclin B2/CDK1 complex also played a negative role in separase activity through inhibitory phosphorylation in meiosis II, which may prevent the incorrect disjunction of sister chromatids.

## Discussion

Proper spatiotemporal regulation of separase activity is important for correct chromosome segregation in oocyte; in turn, the dysregulation of separase activity is likely to cause aneuploidy. In mitosis, separase is known to be inhibited through binding to securin and cyclin B1/CDK1 (Nabti et al., 2008); recently, we have demonstrated that cyclin B2/CDK1 complex inhibits separase activity as well in oocyte meiosis I (Li et al., 2019). In this study, we demonstrate that cyclin B2/CDK1 can also inhibit separase activity in oocyte meiosis II, further elucidating the inhibitory role of cyclin B2 on separase activation and the compensation between cyclin B1 and cyclin B2 in meiosis.

In mouse oocytes, overexpression of either non-degradable securin or non-degradable cyclin B1 prevents segregation of homologous chromosomes in meiosis I (Herbert et al., 2003; Touati et al., 2012), so does overexpression of stable cyclin B2 (Li et al., 2019). Securin is the classical inhibitor of separase (Ciosk et al., 1998), whose degradation is required for anaphase onset mediated by APC/C activity. However, viability and fertility of securin-knockout mice strongly indicated the additional mechanisms on separase regulation independent of securin (Mei et al., 2001; Wang et al., 2001). CDK1 is sufficient for securin-independent inhibition of separase, which is a two-step mechanism with phosphorylation of separase first and stable binding to separase through cyclin B1 (Gorr et al., 2005). Importantly, separase cannot bind both CDK1 and securin simultaneously (Gorr et al., 2005). We stated that cyclin B2 can compensate for cyclin B1 in oocyte meiosis I and interact with separase directly (Li et al., 2018, 2019), demonstrating that CDK1-dependent inhibition of separase is a dual way *via* cyclin B1 and cyclin B2 subunit. In MII eggs, overexpression of either non-degradable securin and cyclin B1 has the ability to prevent the disjunction of sister chromatids (Madgwick et al., 2004); here, we showed that overexpression of stable cyclin B2 also prevented sister chromatid separation and expression of PM-separase rescued the phenotype, suggesting that cyclin B2-associated CDK1 is involved in separase inhibition in oocyte meiosis II as well.

**FIGURE 5:**
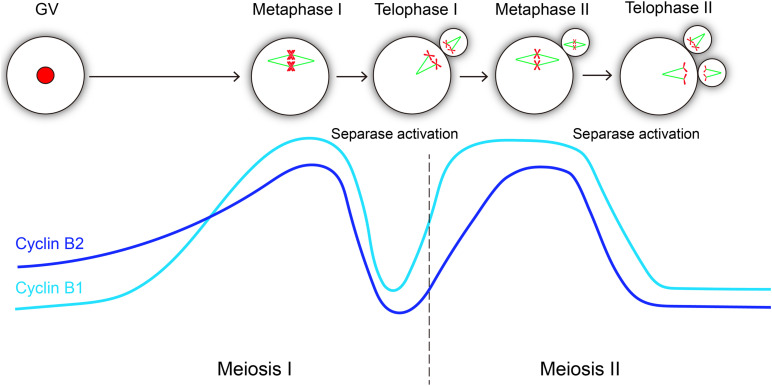
Hypothetical variations of cyclin B1 and cyclin B2 levels and their correlations with separase activity during oocyte meiotic maturation and activation. The level changes of cyclin B1 and cyclin B2 are similar in meiosis progression, both of them reach the peak level at metaphase I and metaphase II, and they exhibit consistent sharp decreases during metaphase-anaphase transitions in meiosis I and meiosis II, which is essential for separase activation. The light blue line represents cyclin B1 protein, and the dark blue line represents cyclin B2 protein.

Cyclin B1 and cyclin B2 are crucial regulatory subunits to activate CDK1 kinase, and cyclin B synthesis and degradation regulates meiotic cell cycle progression in oocyte (Figure 5). In GV-arrested oocytes, the levels of cyclin B2 is significantly higher than those of cyclin B1 (Daldello et al., 2019), which maybe important for meiotic resumption. Remarkably, cyclin B1 is required for meiosis I-meiosis II (MI-MII) transition, while cyclin B2 can compensate for cyclin B1 in meiosis I progression (Li et al., 2018). Cyclin B1 is detectable in the midbody between the oocyte and the first polar body (Huo et al., 2005), suggesting that cyclin B1 is not completely destroyed at the MI-MII transition. Therefore, the residual levels of cyclin B1 should be important for rapid reactivation of CDK1 at the MI-MII transition. Cyclin B2-deleted oocytes can enter metaphase II stage; however, it will probably take more time because many ovulated oocytes remain immature after cyclin B2 deletion. Hence, we speculate that cyclin B2 reaccumulation may facilitate CDK1 reactivation upon MII entry. Given that the expression of stable cyclin B2 prevented the segregation of sister chromatids, it was suggested that cyclin B2 degradation is required for metaphase II-anaphase II transition (Figure 5).

Taken together, our results demonstrate that the cyclin B2/CDK1 complex conservatively inhibits separase activity by inhibitory phosphorylation during meiosis II in mouse oocytes, indicating that cyclin B2 plays its role in separase regulation throughout oocyte meiosis. Above all, given cyclin B2 deletion caused aneuploidy (Daldello et al., 2019), we propose that cyclin B2-associated CDK1 activity is of significance for chromosome segregation during oocyte meiosis. From clinical perspective, our study may provide a potential reference on clinical diagnosis of the patients with failure of oocyte maturation, disorder of oocyte activation, and abnormal chromosome segregation in oocytes.

## Materials and Methods

### Mice

Six- to eight-week-old wildtype female Institute of Cancer Research (ICR) mice were purchased from the Beijing Vital River Laboratory Animal Technology Co., Ltd. and used. All experimental protocols and animal handling procedures were conducted in accordance with the guidelines and procedures approved by the Institutional Animal Care Committee of Institute of Zoology (IOZ), University of Chinese Academy of Sciences (UCAS).

### Oocyte Collection and Manipulation

To collect the matured oocytes (metaphase II oocytes), mice were injected intraperitoneally with 10 U pregnant mare serum gonadotrophin (PMSG) firstly, then the mice were injected intraperitoneally with 10 U human chorionic gonadotropin (HCG) after 48 h; finally, the metaphase II oocytes were collected from the ampulla of oviducts 14–16 h after HCG injection. The oocyte collection was manipulated in M2 medium (M7167, Sigma-Aldrich), and cumulus cells were removed by hyaluronidase (1 mg/ml, Sigma, H3506) treatment. The activated metaphase II oocytes were cultured in prebalanced KSOM medium (MR-121, Sigma-Aldrich).

### Parthenogenetic Activation

Oocyte parthenogenetic activation was induced by 10 mM strontium chloride (SrCl2, Sigma) in Ca^2+^-free CZB medium for 4–6 h. After parthenogenetic activation, eggs were transferred into KSOM (MR-106, Millipore) for further culture; subsequently, the rate of pronuclear formation was counted.

### Chromosome Spreads

Chromosome spreads were performed according to the procedure described previously (Li et al., 2019). Briefly, after proper removal of zona pellucida, the oocytes were washed in prewarmed M2 medium for a brief recovery; subsequently, the oocytes were exposed to spread solution (1% paraformaldehyde in distilled H_2_O (pH 9.2) containing 0.15% Triton X-100 and 3 mM dithiothreitol) on a clean glass slide, as previously reported (Hodges and Hunt, 2002). After drying slowly in a half-open humidified chamber at room temperature (RT), the fixed oocytes were blocked with 2% BSA in PBS for 1 h at RT. For immunofluorescent staining, the oocytes were then incubated with primary antibodies overnight at 4°C. After three washes (10 min each wash), the slides were then incubated with corresponding secondary antibody for 2 h at RT. Primary human anti-ACA (anticentromere antibody) antibody (1:50; 15–234, Antibodies Incorporated) was used for detecting centromeres with a corresponding secondary antibody conjugated with Alexa Fluor Cy5 (709-175-149, Jackson ImmunoResearch).

### Preparation of cRNAs and Microinjection

To make the non-degradable cyclin B2 mutant, the Destruction-box (N-terminal 50 amino acids) of murine cyclin B2 was deleted and cloned into a pcDNA3.1-Venus vector and a pCS2(+)-Myc vector, respectively, as described previously (Li et al., 2019). The murine WT-separase and PM-separase (S1121A) constructs were cloned into pCS2 vector as reported previously (Touati et al., 2012; Li et al., 2019). The separase sensor was cloned into pGEMHE vector. The cRNAs were prepared using SP6 or T7 mMessage mMachine (Ambion), respectively, then purified with RNeasy kit (74004, Qiagen), dissolved in nuclease-free water, and stored at −80°C. Microinjection was performed with a Nikon operating system.

### Time-Lapse Confocal Live-Cell Imaging

Live-cell imaging was performed using a PerkinElmer Ultra VIEW-VoX confocal imaging system equipped with a CO_2_ incubator chamber (5% CO_2_ at 37°C). Digital time-lapse images (30 z-slices with 2 μm spacing) were acquired using a 20 × 0.75 objective lens, and Volocity 6.0 software was used for image acquisition. Injected metaphase II oocytes were incubated in KSOM medium for time-lapse imaging. To track the signals for Δ50cyclin B2-Venus and mCherry-H2B, images were taken at the maximum speed. To track the change of separase sensor, images were also taken at the maximum speed.

### Calcium Oscillation Detection

Oocyte calcium oscillations was detected with a frequently used fluorescent probe Fluo-4AM (1 μm, Beyotime) under PerkinElmer Ultra VIEW-VoX confocal time-lapse imaging system; the excitation wavelength of laser is 488 nm. Real-time images and signals were collected twice per minute.

### Statistical Analysis

Statistical analysis was processed by Student’s *t*-test using Prism 5 (GraphPad Software). Images were analyzed with ImageJ (National Institutes of Health) and Photoshop CS5 (Adobe) software and composed by Illustrator CC5 (Adobe) software.

## Data Availability Statement

The raw data supporting the conclusions of this article will be made available by the authors, without undue reservation.

## Ethics Statement

The animal study was reviewed and approved by the Institutional Animal Care Committee of Institute of Zoology (IOZ), University of Chinese Academy of Sciences (UCAS).

## Author Contributions

JL, Q-YS, and W-PQ conceived and designed the project. JL prepared and performed the experiments. JL and Q-YS analyzed the data and prepared the manuscript. H-YZ and FW provided technical support. All authors approved the final version of the manuscript.

## Conflict of Interest

The authors declare that the research was conducted in the absence of any commercial or financial relationships that could be construed as a potential conflict of interest.
